# Linear Dispersal of the Filth Fly Parasitoid *Spalangia cameroni* (Hymenoptera: Pteromalidae) and Parasitism of Hosts at Increasing Distances

**DOI:** 10.1371/journal.pone.0129105

**Published:** 2015-06-10

**Authors:** Erika T. Machtinger, Christopher J. Geden, Norman C. Leppla

**Affiliations:** 1 Entomology and Nematology Department, University of Florida, PO Box 110620, Gainesville, Florida, 32611, United States of America; 2 USDA-ARS, Center for Medical, Agricultural, and Veterinary Entomology, 1600 S. W. 23^rd^ Drive, Gainesville, Florida, 32608, United States of America; Institute of Zoology, CHINA

## Abstract

Release of parasitic wasps (Hymenoptera: Pteromalidae) as biological control agents for house flies and stable flies in livestock confinements has had variable success. In part, this may reflect a lack of knowledge regarding the optimal distance to be used between parasitoid release stations. In the current study, we assessed the effect of linear distance on host parasitism by the wasp *Spalangia cameroni* Perkins. In open fields at distances ranging from 1 m to 60 m from a central point, house fly puparia were placed in a mixture of pine shavings soiled with equine manure, urine, and alfalfa hay. Releases of *S*. *cameroni* then were made using a 5:1 host: parasitoid ratio. Host pupae were parasitized at all distances, with the highest rate of total parasitism (68.9%) recorded ≤ 5 m from the release site. Analyses of results using non-linear and linear models suggest that *S*. *cameroni* should be released in close proximity to host development areas. Additionally, releases may not be suitable in pasture situations where long-distance flight is required for control. However, further testing is needed to examine the effect of density-dependent dispersal and diffusion of *S*. *cameroni*.

## Introduction

House flies (*Musca domestica* Linnaeus) and stable flies (*Stomoxys calcitrans* ((L.)) are a major nuisance in livestock facilities. High numbers of flies can cause loss of condition to livestock [1–3] and raise concerns of human and animal safety; e.g., house flies in particular transmit many pathogens that cause disease [4–5]. Control of these flies has been primarily with insecticides, but high levels of insecticide resistance in fly populations [6–10] has increased the need for alternative control methods. One method is the inundative release of pupal parasitoids (Hymenoptera: Pteromalidae), particularly species of *Muscidifurax* and *Spalangia*, as biological control agents.

The use of pupal parasitoids has been successful in some evaluation studies [11–16]. However, in some cases, significant fly control has not been achieved [17–21]. The lack of efficacy in some of these trials may be a consequence of insufficient knowledge to develop release protocols, including optimal distance between release sites or stations. Two common parasitoid release methods are scattering parasitized host puparia on the ground near fly development sites [22–24] and allowing parasitoids to disperse from artificial devices placed in or near known fly development areas [16, 21, 22–23]. In some release studies, the distance between release stations is not given or the method of release is not disclosed.

Knowing the distance a parasitoid will disperse from a release point and subsequently parasitize hosts is important for optimizing releases in augmentation programs. However, little attention has been given to the dispersal distance of pteromalid parasitoids. Dispersal evaluations of *Muscidifurax* spp. have been variable depending on the study location and species. *Muscidifurax zaraptor* Kogan and Legner was found to disperse no more than 8 m by Pawson and Petersen [25] and *M*. *raptor* Girault and Saunders was recovered less than 30 m from a release point in a dairy facility [26]. Parasitism by *M*. *raptorellus* Girault and Saunders was not observed greater than 6 m away from a release point in a high rise poultry unit, regardless of release numbers [27]. However, greater dispersal distances of 22.5 m, 48 m, and 100 m were reported by Lysyk [28], Petersen and Cawthra [13], and Floate et al. [29], respectively, in cattle feedlots. Dispersal of *Spalangia cameroni* Perkins, one of the most common commercially available filth fly pupal parasitoids, was approximately 3 m from a release point in indoor swine facilities [30], though structural complexity of the stable and cool temperatures were proposed as impediments to movement. The effect of host distance from a release point on dispersal and subsequent parasitism in pasture environments has not been studied for *S*. *cameroni*.

The necessary number, location, and spacing of release points depends on the dispersal ability of the parasitoids. Models to predict parasitoid movement can be developed from dispersal data [31–35]. The primary aim of this study was to determine the effect of linear distance on parasitoid dispersal and parasitism of house fly hosts located in equine waste and bedding material. A second objective was to test dispersal models to better understand parasitoid movement after release.

## Materials and Methods

The host habitat substrate used in this dispersal study was a mixture of pine shavings (0.1 to 0.3 cm long) soiled with horse manure and urine and containing trace amounts of alfalfa (*Medicago sativa*) hay collected 72 h after defecation as described in Machtinger et al. [36]. This material was collected from horse stalls on a private equine farm in Ocala, Florida with permission from the owner. This material was found to be a highly attractive substrate for house fly oviposition [37] and attractive to *S*. *cameroni* for host-seeking [37]. The substrate was held at -18°C for a minimum of 1 week prior to use to kill any existing arthropods.

Immature house flies, obtained from the USDA-ARS, Center for Medical, Agricultural and Veterinary Entomology (CMAVE) insecticide-susceptible colony, were reared on the diet described in Hogsette [38]. In brief, the diet contained 5 L of fly diet mix, (50% wheat bran, 30% alfalfa meal and 20% fine corn meal) and 3.75 L of water. House fly larvae were separated *en masse* from rearing media 1-d from expected pupariation and then approximately 2000 as determined by weight were scattered over the surface of the 11 L of substrate (depth of 17 cm) in each of four plastic 45 L bins (55 cm-long x 25 cm-wide x 33 cm-high). The outside walls of each bin were coated with Insect-a-slip (ethylene tetrafluoroethylene) (BioQuip, Inc., Rancho Dominguez, CA) to minimize potential insect predation. Each bin had a lid (55 cm-long x 25 cm-wide) with standard window screening (fine 16/14 mesh) to exclude vertebrate predators. Bins were held for 48 h at 23^°^C before field trials to give larvae time to pupariate,

*Spalangia cameroni* used for parasitoid releases were obtained from a laboratory colony maintained on house fly hosts for about 24 generations prior to the study. This colony was established in 2010 from a large source population on a dairy farm in Gilchrist County, FL collected with the owner’s permission. One-day-old females were separated from males while anesthetized briefly on a cooling table (approximately 4^°^C) and then counted into groups of 400 and placed in 1-oz (30 cm^3^) plastic cups. Females were held without hosts at 25^°^C for 12 h and then released at a 5:1 host to parasitoid ratio (~2000 house fly puparia and 400 female parasitoids).

Releases were conducted in the summer of 2012 in two fields planted with bahai grass (*Paspalum notatum*) at the University of Florida (Gainesville, FL) with permission from the University. These fields did not house livestock and were at least 2 km from livestock facilities, which minimized the risk of extraneous parasitism by local populations of parasitoids. Each field was divided into two plots. A parasitoid release station was located in each plot, separated by a distance of at least 100 m. Releases in two plots were conducted south to north, one plot releases were north to south, and releases in one plot were southeast to northwest. Bins with hosts were placed in the field at each of the following treatment distances from a stationary release point: 1 m, 5 m, 10 m, 20 m, 30 m, and 60 m. Dispersal was tested one distance at a time. Bin placement at each distance and each plot was randomized using a random number generator. Initially, bins were placed in the field before any parasitoids were released to estimate natural background parasitism. Each bin was protected from heavy rain and direct sunlight with a plywood roof positioned 30 cm above the top of the bin.

Lids were removed and the plastic cup holding parasitoid females was placed into a PVC pipe (30-cm-long and 5-cm-diameter) with nine 3-cm holes covered with window screening and covered on both ends with a 5-cm-diameter PVC cap. The PVC pipes were suspended 1-m from the ground to avoid predation. Bin placement and parasitoid releases were conducted at dawn (approximately 6 am). Releases occurred at one plot in each field (two release plots per week) with the second plot on each field acting as a control with a bin with substrate and hosts but no parasitoid releases (two control plots per week). Releases occurred weekly and were conducted once at each of the four plots for each of the six distances (4 replicates total per distance). Releases began June 1, 2012 and continued until August 17, 2012. Ambient temperature ranged from 27^°^C to 35^°^C throughout the study, consistent with Florida summers. Bins were collected 3 d after parasitoid release and puparia sifted through 2.38 mm mesh (no.8 US Standard sieve) from the substrate. Puparia were held for parasitoid emergence in ventilated 9-oz plastic cups for a minimum of 8 weeks at 27^°^C and 80% RH. Emerged parasitoids were identified to species by the authors.

Data on recovered puparia were analyzed with Analysis of Variance (ANOVA) using JMP v. 11 (SAS Institute Inc., Cary, NC 2013). Separate analyses were conducted for two response variables: (1) percentage parasitism; i.e., the number of puparia producing a parasitoid divided by the total number of recovered puparia, and (2) residual host mortality or unexplained host mortality; i.e., the number of puparia not producing a fly or parasitoid divided by the number of recovered puparia [37]. Parasitoid progeny production was corrected for background parasitism by subtracting the mean background parasitism by the recovered progeny. Though the use of percentage parasitism as a metric for analysis of dispersal has limitations (*i*.*e*., parasitoids may disperse but not parasitize hosts), because the recovery of these small parasitoids after release is nearly impossible percentage parasitism was determined to be the most appropriate quantitative measurement of dispersal for this species. Parasitoid progeny and residual host mortality counts were normalized with a log transformation; values in tables and text are reported as original units. Means calculated for each distance were separated with Tukey’s HSD test for comparison (α = 0.05).

Data on recovered parasitoid progeny were tested in seven dispersal models in SAS v. 9.2 (SAS Institute Inc., Cary, NC 2008). PROC REG was used for initial parameter estimates; using the first derivative of each model these estimates were used to initialize variables in a subsequent analysis using PROC NLIN to test model fit. Five non-linear models were evaluated from three studies analyzing insect dispersal [33–35] and one linear model (Model 6):

Model 1: $N\;=\;e^{\left(a+bx^{-1}\right)}$

Model 2: $N\;=\;e^{\left(a+blnx\right)}$

Model 3: $N\;=\;e^{\left(a+b\surd x\right)}$

Model 4: $N\;=\;e^{\left(a+bx\right)}$

Model 5: $N\;=\;e^{\left(a+bx^{2}\right)}$

Model 6: $\log N\;=\;e^{\left(X\right)}$

In each of these models, N = number of individuals dispersing as measured by the number of progeny recovered from the hosts, x = distance, and a and b are constants where a is the y-intercept and b is the slope of the x coefficient. These models were selected to determine if *S*. *cameroni* followed similar patterns of dispersal found in other parasitic Hymenoptera. The diffusion component of dispersal was not included because the experimental design was entirely linear and therefore dispersal of parasitoids away from the attractive substrate was not measured. Models were compared based on the coefficient of determination (r^2^) as well as fit of observed verses predicted values.

## Results

Background parasitism measured from both experimental fields prior to the study was minimal, with only 0.06% of puparia being parasitized by two species, *S*. *cameroni* and *S*. *endius* Walker. Combined across all experimental distances, 47,686 puparia were recovered from the shavings and equine manure substrate (Table 1), of which 7,594 (15.9%) produced parasitoids. Of the 8,815 puparia recovered at 1 m, 46.9% were parasitized, which accounted for 54.4% of the total observed cases of parasitism. At 5 m, only 13.1% of the recovered puparia were parasitized. Parasitism declined only slightly from 10 to 30 m (ranging from 9.9 to 8.8%), but dropped to 3.2% at 60 m.

**Table 1:** Recovery of house fly puparia, percentage parasitism, and parasitoid species at distances of 1 to 60 m from points of mass-release with female *Spalangia cameroni*

Distance (m)^a^	Total Puparia Recovered	Total Emerged Parasitoids	Parasitism (%)	*Spalangia cameroni* species recovery^c^	*Spalangiaendius* species recovery^c^	*Spalangia nigroaenea* species recovery^c^	*Pachycrepoideus vindemiae* species recovery^c^
1	8,815	4,133	46.9	96.2	2.2	0.0	1.7
5	8,410	1,104	13.1	94.2	5.3	0.0	0.5
10	8,663	857	9.9	99.1	0.0	0.1	0.0
20	7,409	666	9.0	96.2	3.8	0.0	0.0
30	6,724	592	8.8	100.0	0.0	0.0	0.0
60	7,665	242	3.2	100.0	0.0	0.0	0.0
Total	47,686	7,594	15.9^b^	97.7^b^	1.9^b^	0.02^b^	0.4^b^

^a^Four replicates were conducted for each distance (n = 400 female parasitoids per release, 1600 total)

^b^Numeric values are presented as the mean of the column.

^c^mean % of total recovered for each distance

Four species of parasitoids were recovered during the study. As expected, *S*. *cameroni* accounted for 97.7% of all parasitoid species recovered (Table 1). *Spalangia endius* was the second most common parasitoid, comprising 1.9% of total recoveries. *Pachycrepoideus vindemiae* Rondani accounted for 0.4% of total progeny and a single specimen of *Spalangia nigroaenea* Curtis was recovered.

Parasitoid progeny recovery from 1 m was significantly higher than recovery at all other distances (Table 2). Average progeny numbers declined steadily from 5 to 30 m. At 60 m, the number of progeny recovered was less than half of that recovered at 30 m, though progeny recoveries at this distance did not differ statistically from the closer distances. Parasitoid progeny recovered from control bins was low, ranging from 1.1 ± 0.72 (1 m) to 12.0 ± 12.0 (10 m). Control bins were not statistically different from each other and did not follow a pattern. Residual host mortality was similar from 1 to 10 m, and significantly higher than the remaining distances, ranging from 487.0 (± 44.0) recovered at 5 m to 535.0 (± 63.5) at 10 m. Combining parasitism and residual host mortality, the mean total host mortality was 68.0% at 1 m, dropping to 32.3 and 34.6% at 5 m and 10 m, respectively. Residual host mortality dropped after 10 m to similar levels observed in control bins. None of the residual host mortality differed significantly among control bins.

**Table 2:** Comparison of recovered parasitoid progeny and residual host mortality ($\bar{x}$ ± SE) in treatment and control bins after releases of *Spalangia cameroni* at a 1:5 parasitoid:host ratio.

Distance (m)^a^	Emerged Parasitoids ($\bar{x}$ ± SE)^b^		Residual Host Mortality ($\bar{x}$ ± SE)^a^	
	Experiment bins^c^	Control Bins	Experiment bins	Control Bins
1	983.3 ± 178.0a	1.1 ± 0.7a	515.3 ± 43.9a	150.5 ± 30.3a
5	276.0 ± 65.6b	10.0 ± 7.1a	487.0 ± 44.0a	207.3 ± 31.7a
10	214.3 ± 37.6b	12.0 ± 12.0a	535.0 ± 63.5a	196.3 ± 45.2a
20	166.5 ± 9.2b	6.0 ± 4.0a	134.0 ± 21.8b	194.0 ± 39.5a
30	148.0 ± 31.6c	5.4 ± 4.7a	157.0 ± 8.6b	176.0 ± 16.0a
60	60.5 ± 19.3c	5.9 ± 3.4a	176.0 ± 24.2b	197.0 ± 20.2a

House fly puparia were provided in pine shavings and equine manure in plastic bins at distances ranging from 1 to 60 m from a release point.

^a^Four replicates were conducted for each distance (n = 400 female parasitoids per release, 1600 total)

^b^ Means in a column followed by the same letter are not significantly different (Tukey’s HSD test, α = 0.05) and values were log transformed.

^c^ Experiment bins were subjected to parasitoid releases while control bins were not. Experiment bins were tested once in each of the four field plots for each distance with a corresponding control bin.

**Fig 1:**
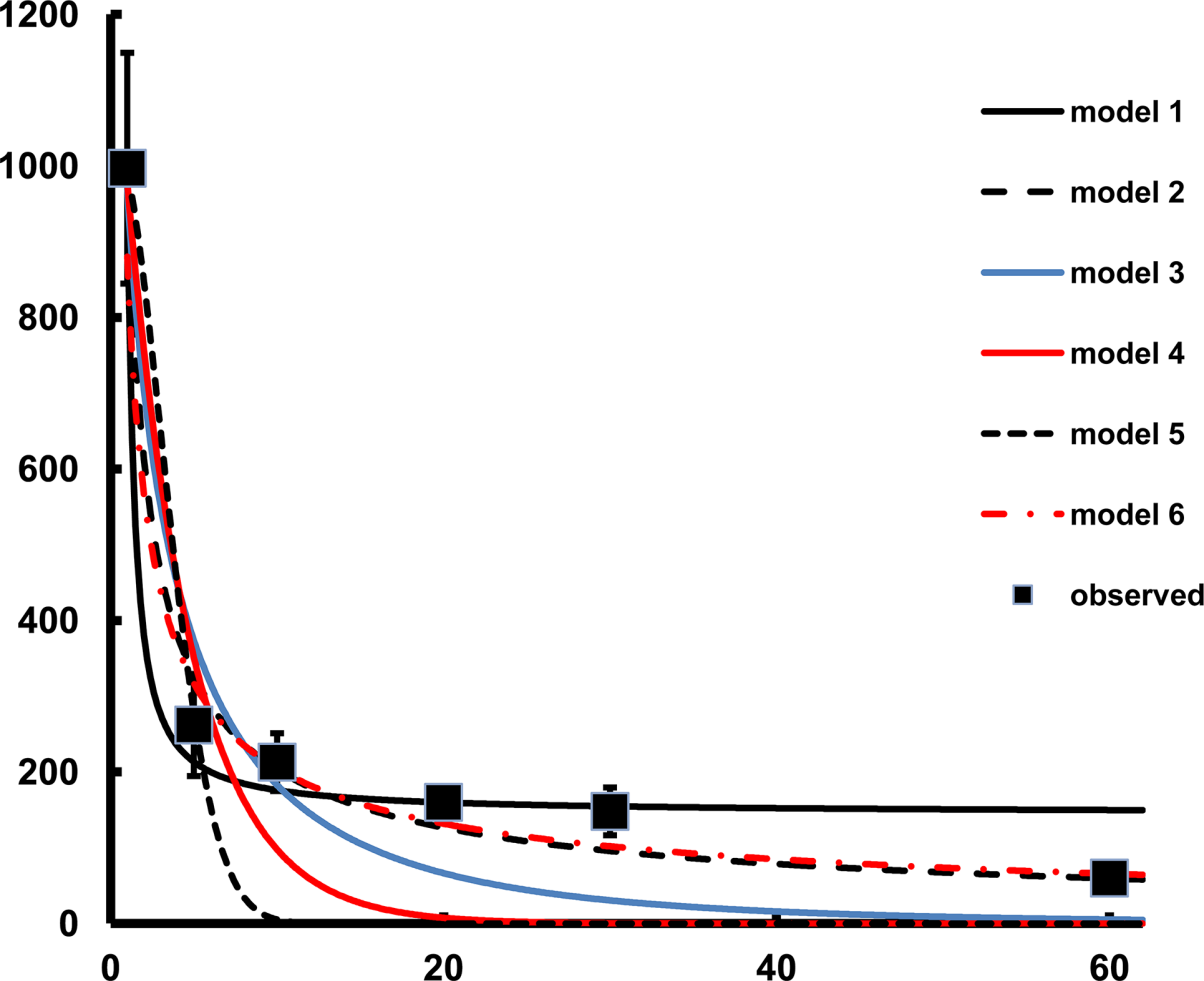
Observed and predicted dispersal of *Spalangia cameroni* in open fields analyzed using recoveries of progeny from house fly hosts with equine substrate and at a 1:5 parasitoid: host ratio. Four replicate releases were conducted for each distance (n = 400 female parasitoids per release, 1600 total)

Three of the six tested models closely fitted the data. Model 1 and Model 2 had R^2^ values of 0.917 and 0.920, respectively. However, although Model 6 resembled Model 2, the R^2^ value was 0.771 (Table 3). Models 3, 4, and 5 did not fit the data well, severely underestimating dispersal. The parasitoid progeny recovery predictions were accurate with Model 1 until 60 m where this model overestimated the predicted recovery (Fig 1). Based on the data, the model that best fit the data was Model 2. This model took the following form: Model 2: $N\;=\;e^{\left(a+blnx\right)}$

**Table 3:** Parameter estimates and coefficients of determination for six models tested and compared to parasitoid progeny recovered at six distances after releases of *Spalangia cameroni*.

Model	Parameter a (estimated ± SE)	Parameter b (estimated ± SE)	R^2^	F
Model 1	4.977 (0.197)	1.930 (0.211)	0.917	254.27
Model 2	6.780 (0.203)	-0.634 (0.074)	0.920	263.67
Model 3	6.741 (0.225)	-0.369 (0.491)	0.895	195.39
Model 4	6.059 (0.183)	-0.038 (0.006)	0.624	36.56
Model 5	5.703 (0.174)	-0.001 (0.001)	0.566	29.99
Model 6	6.780 (0.203)	-0.634 (0.074)	0.771	73.84

## Discussion

Knowing how far *S*. *cameroni* can disperse can improve release strategies when using this parasitoid for filth fly management. We found that *S*. *cameroni* was capable of dispersing and parasitizing hosts at least 60 m from a release site, although the highest rate of total parasitism (68.9%) was recorded from ≤ 5 m from the release site. These data suggest that *S*. *cameroni* generally tends to parasitize hosts close to a release site and our objectives to assess linear dispersal and model parasitoid movement were met, however, our study was not designed to account for the effects of spatial parasitoid density or diffusion in relation to dispersal distance from a release site [35, 39]

Information on dispersal of filth fly pupal parasitoids is limited, possibly because of the inherent difficulty in tracing the dispersal of small organisms [40]. In other studies with *S*. *cameroni*, dispersal was similarly limited [30]. Similar short-range dispersal, primarily from 2 m to 8 m, was observed with other filth fly pupal parasitoids in cattle feed lots and poultry caged-layer facilities [25, 27–28], and similar declines in parasitism by distance were found.

The dispersal range recorded may have been a result of environmental conditions, density-dependent factors, or olfactory cues. Though environmental conditions including wind direction, wind speed, and rainfall may influence parasitoid dispersal, releases were not conducted during anomalous weather patterns and, in this case releases, were conducted over three days and thus these specific conditions were not recorded. Dispersal from a release point can be affected by the density of parasitoids [41]. Minimal numbers of mated adult females were released to accurately estimate the distance traveled by an individual to parasitize a host. The observed dispersal to hosts primarily close to release sites may be explained by the low numbers of *S*. *cameroni* released. However, significantly lower levels of parasitism by *M*. *zaraptor* and *M*. *raptorellus* were observed farther from release stations, regardless of high parasitoid release rates [13, 25, 27–28]. Olfactory cues are important for parasitoid host-seeking [42]. Parasitoids use volatiles, e.g. kairomones [42], emitted from fungi and bacteria in habitats [43] and from different host development stages [44] to locate suitable hosts. Maximum dispersal distance may extend past the observed 60 m and host parasitism by *S*. *cameroni* may be greater farther from release sites in areas with established waste accumulation, unlike our ephemeral bins with substrate and hosts. However, the chemical cues associated with directed dispersal towards host habitat may be limited in pasture where host development habitat is intermittent and ephemeral. This may suggest that this species is not suitable for single-site releases for fly control in pasture unless fly development areas are identified prior to release or releases stations cover areas uniformly. Further examination of parasitoid dispersal with a spatial model could generate improved data on parasitoid behavior after release.

Of the six tested models, Model 2 accurately predicted dispersal of *S*. *cameroni*. Due to time constraints, four replicates were conducted for each distance and while model predictions were supported by recoveries from the field, further replication and analysis might improve accuracy. Similar to *S*. *cameroni*, the dispersal patterns of several *Cotesia* spp. (Hymenoptera: Brachonidae) parasitoids have been fit to non-linear models [33–34] with similar maximum dispersal ranges observed. The recoveries of other species of pteromalid parasitoids at short-range distances from release points further supports the results of this model for the dispersal capability of *S*. *cameroni*.

Pupal parasitoids can be an effective and environmentally sound alternative to chemical fly control. With a better understanding of the behavior of parasitoids after release, techniques to introduce parasitoids into a system as biological control agents can be refined to optimize parasitism of fly pests. *Spalangia cameroni* is a widespread, common, and commercially available pupal parasitoid. Though this species does not appear to disperse far from a release site, the results presented herein show that even low numbers of parasitoids can parasitize a significant number of hosts in a short period of time when released in close proximity. Further research is needed to investigate the impact of density-dependent and directional dispersal. However, based on these results, the efficacy of control using this species will likely be greatest if parasitoids are released immediately adjacent to targeted fly development habitats. Additionally, releases should be coupled with an integrated pest management program that emphasizes cultural control practices to reduce fly breeding.
